# Supplementary material for: #Caremongering: A community-led social movement to address health and social needs during COVID-19
Source: PLoS One. 2021 Jan 14;16(1):e0245483. doi: 10.1371/journal.pone.0245483 (PMC7808573; doi:10.1371/journal.pone.0245483)
Supplement: S1 Table — (DOCX) [file pone.0245483.s001.docx]

**S1 Table. Caremongering Group Data**

| **Group Name** | **City** | **Province** | **Date group created** | **# of Members Apr 12 2020** | **# of Members May 4 2020** | **# of Members June 9** | **# of Members Sept 3 2020** | **Average posts/day per 100 members (May)** |
| --- | --- | --- | --- | --- | --- | --- | --- | --- |
| CareMongering - TO: TO Community Response to COVID19 | Toronto | ON | 3/12/2020 | 22,845 | 24,822 | 25,104 | 25,000 | 1.25 |
| Caremongering-HFX: Halifax-Area Community Response to COVID19 | Halifax | NS | 3/13/2020 | 11,245 | 11,794 | 11,594 | 11,414 | 0.76 |
| CareMongering-KW: Kitchener-Waterloo Community Response to COVID 19 | Kitchener | ON | 3/13/20 | 8,085 | 8,745 | 8,523 | 8,403 | 0.91 |
| COVID-19 Community Care Ottawa | Ottawa/Gatineau | ON | 3/13/20 | 6,293 | 6,840 | 6,866 | 6,811 | 0.58 |
| CareMongering - HamOnt: Hamilton Community Reponse to COVID19 | Hamilton | ON | 3/13/2020 | 6,271 | 6,997 | 7,070 | 7,011 | 1.14 |
| YXE Community Response COVID 19 | Saskatoon | SK | 3/16/2020 | 5,593 | 6,810 | 6,980 | unable to locate | 1.91 |
| Be The Light- Annapolis Valley 2020 Pandemic Community | Kentville (Annapolis Valley) | NS | 3/15/2020 | 5,538 | 5,998 | 6,093 | 6,094 | 3.50 |
| Burlington Together (CareMongering BURLINGTON) | Hamilton (Burlington) | ON | 3/15/2020 | 5,532 | 6,287 | 7,174 | 8,833 | 2.23 |
| CareMongering - YGK/Kingston | Kingston | ON | 3/13/2020 | 4,849 | 5,232 | 5,247 | 5,324 | 1.15 |
| Caremongering Guelph- Leaning into Community During COVID19 | Guelph | ON | 3/13/20 | 4,597 | 5,229 | 5,329 | 5,382 | 5.35 |
| CareMongering-Ott: Ottawa Community Response to COVID19 | Ottawa/Gatineau | ON | 3/13/20 | 4,442 | 4,774 | 4,679 | 4,562 | 0.63 |
| Caremongering- Saskatoon | Saskatoon | SK | 3/16/2020 | 4,325 | 4,671 | 4,698 | 4,659 | 1.07 |
| CareMongering-Niagara, Ontario: Niagara Community Response to COVID-19 | St. Catharines- Niagara | ON | 3/13/2020 | 4,074 | 4,304 | 4,283 | 4,149 | 0.14 |
| Caremongering - Kamloops | Kamloops | BC | 3/17/2020 | 3,532 | 4,020 | 4,144 | 4,201 | 0.25 |
| Swift Current Pandemic Suppport Network | Swift Current | SK | 3/14/2020 | 3,459 | 3,858 | 3,941 | 4,395 | 1.30 |
| Caremongering-PEI: Response to COVID-19 | Charlottetown | PEI | 3/14/2020 | 3,454 | 3,642 | 3,651 | 3,654 | 0.25 |
| Caremongering London, ON - Community & ACTIVIST Responses to Covid-19 | London | ON | 3/12/2020 | 3,157 | 3,534 | 3,402 | 3,363 | 3.11 |
| Orangeville And Area CAREMONGERING | Orangeville | ON | 3/14/2020 | 3,042 | 3,501 | 3,610 | 3,671 | 3.14 |
| Caremongering in Fredericton: Social Distancing Support & Information | Fredericton | NB | 3/15/2020 | 3,005 | 3,183 | 3,191 | unable to locate | 0.94 |
| Capers helping capers during COVID-19 #caremongering Cape Breton | Glace Bay (Cape Breton) | NS | 3/15/2020 | 2,966 | 3,208 | 3,201 | 3,199 | 0.62 |
| CareMongering Windsor/Essex | Windsor | ON | 3/13/2020 | 2,745 | 3,213 | 3,300 | 3,377 | 1.24 |
| Caremongering - Norththumberland County Community Response to COVID 19 & Beyond | Cobourg | ON | 3/14/2020 | 2,567 | 2,839 | 2,851 | 2,818 | 1.76 |
| Amherstburg (Caremongering (COVID - 19) Related Topics | Windsor - Essex (Amherstberg) | ON | 3/18/2020 | 2,138 | 2,622 | 2,737 | 2,943 | 6.48 |
| Caremongering Happy Valley-Goose Bay | Happy Valley- Goose Bay | NFL | 3/17/2020 | 1,708 | 1,817 | 1,822 | 1,838 | 0.55 |
| Caremongering - SaintJohn/Roth/Quispam/GB/Westfield Area Response to COVID 19 | Saint John | NB | 3/16/2020 | 1,690 | 1,776 | 1,770 | 1,737 | 0.23 |
| Sarnia Caremonger Group | Sarnia | ON | 3/17/2020 | 1,689 | 1,890 | 1,895 | 1,878 | 0.53 |
| Caremongering Kelowna | Kelowna | BC | 3/17/2020 | 1,659 | 1,815 | 1,834 | 1,773 | 0.55 |
| Caremongers Whitby | Toronto (Whitby) | ON | 3/14/2020 | 1,651 | 2,065 | 2,207 | 2,339 | 1.94 |
| Caremongering- Sudbury, ON | Sudbury | ON | 3/17/2020 | 1,610 | 1,749 | unable to locate | unable to locate | 0.11 |
| Red Deer COVID-19 Volunteers | Red Deer | AB | 3/13/2020 | 1,573 | 1,693 | 1,663 | 1,626 | 0.30 |
| CareMongering-NB: North Bay, ON Community Response to COVID19 | North Bay | ON | 3/18/20 | 1,530 | 1,723 | 1,720 | 1,690 | 1.16 |
| Caremongering- Regina/YQR | Regina | SK | 3/16/2020 | 1,524 | 1,645 | 1,650 | 1,625 | 0.12 |
| CareMongering Centre Wellington- CW Community Response to COVID19 | Fergus | ON | 3/14/2020 | 1,472 | 1,591 | 1,601 | 1,592 | 1.26 |
| Caremongering Oshawa and Clarington | Oshawa | ON | 3/12/20200 | 1,420 | 1,774 | 1,828 | 1,818 | 2.82 |
| Caremongers Windsor-West Hants in response to COVID-19 pandemic | Windsor | NS | 3/17/2020 | 1,251 | 1,451 | 1,475 | 1,537 | 2.07 |
| CareMongers-North Bay and Area | North Bay | ON | 3/17/2020 | 1,228 | 1,464 | 1,643 | 1,648 | 1.37 |
| Caremongering YK | Yellowknife | NT | 3/16/2020 | 1,206 | 1,505 | 1,526 | 1,574 | 0.66 |
| Covid-19 Communinty Support/ Help for Bridgewater and all surrounding areas | Bridgewater | NS | 3/16/2020 | 1,166 | 1,233 | 1,218 | 1,214 | 0.32 |
| Caremongering YVR - Vancouver Community Response to COVID19 | Vancouver | BC | 3/14/2020 | 1,097 | 1,214 | 1,213 | unable to locate | 0.49 |
| Care-mongering Toronto - Arts / Live Events Industry | Toronto | ON | 3/13/2020 | 996 | 1,071 | 1,079 | 1,083 | 0.19 |
| Caremongers- Sarnia | Sarnia | ON | 3/17/20 | 988 | 1,129 | 1,129 | 1,086 | 0.89 |
| Caremongering Renfrew County and Area | Pembroke | ON | 3/16/2020 | 976 | 1,071 | 1,074 | 7,902 | 0.37 |
| CareMongering - Abbotsford: Community Response to COVID 19 | Abbotsford | BC | 3/16/2020 | 943 | 1,035 | 1,037 | 1,026 | 0.97 |
| Caremongering in Truro: Community Social Distancing and Support | Truro | NS | 3/15/2020 | 894 | 1,002 | 998 | 989 | 1.00 |
| Ottawa Caremongering- COVID19 | Ottawa/Gatineau | ON | 3/16/20 | 892 | 963 | 988 | 567 | 0.83 |
| Caremongering Yorkton and area | Yorkton | SK | 3/18/2020 | 873 | 948 | 949 | 947 | 0.53 |
| CareMongering- YEG | Edmonton | AB | 3/14/2020 | 849 | 899 | 888 | 882 | 0.44 |
| CareMongering YYC: YYC Community Response to COVID-19 | Calgary | AB | 3/16/2020 | 828 | 885 | 875 | 849 | 0.68 |
| #Caremongering/ Georgina Helps | Keswick-Elmhurt Beach | ON | 3/14/2020 | 777 | 871 | 885 | 870 | 0.57 |
| CareMongering - WINNIPEG Community Response to COVID19 | Winnipeg | MB | 3/16/2020 | 770 | 871 | 875 | 891 | 1.15 |
| Caremongering Inuvik | Inuvik | NT | 3/17/2020 | 770 | 890 | 908 | 909 | 1.12 |
| CareMongering Peel: Peel Community Response to COVID-19 | Toronto (Peel Community) | ON | 3/15/2020 | 750 | 858 | 890 | 927 | 0.70 |
| Caremongering- West Island Montreal | Montreal | QB | 3/16/2020 | 656 | 733 | 732 | 737 | 0.68 |
| CareMongering - Orillia | Orillia | ON | 3/14/2020 | 652 | 776 | 779 | unable to locate | 1.29 |
| Caremongering Vancouver Island: Community Response to COVID19 | Vancouver | BC | 3/17/2020 | 631 | 687 | 684 | 660 | 0.44 |
| CareMongering-Brantford: Brantford Community Response to COVID 19 | Brantford | ON | 3/14/2020 | 613 | 753 | 797 | 804 | 1.33 |
| Caremongering St. John's NL | St. John's | NFL | 3/17/2020 | 545 | 574 | 569 | 685 | 1.39 |
| Caremongering-Kentville-Wolfville Area Community Response to COVID-19 | Kentville | NS | 3/14/2020 | 520 | 543 | 548 | 548 | 0.37 |
| Caremongering- Montreal | Montreal | QB | 3/16/2020 | 444 | 490 | 492 | 489 | 0.41 |
| CareMongering - Barrie: Community Response to COVID - 19 | Barrie | ON | 3/18/2020 | 432 | 486 | 483 | 473 | 0.41 |
| CareMongering-YT : Yukon Community Response to COVID-19 | Whitehorse | YT | 3/14/2020 | 394 | 445 | 449 | 447 | 0.45 |
| Caremongering-Pointe-Claire QC | Montreal | QB | 3/17/2020 | 388 | 433 | 437 | 446 | 2.31 |
| CareMongering- Town of the Blue Mountains & Area | Collingwood | ON | 3/17/2020 | 375 | 432 | 449 | 456 | 2.31 |
| CareMongering Edmonton, AB & Area: Response to COVID-19 | Edmonton | AB | 3/18/2020 | 355 | 392 | 392 | 390 | 1.02 |
| Caremongering - Wasaga Beach | Wasaga Beach | ON | 3/28/2020 | 352 | 474 | 495 | 494 | 1.48 |
| Fighting COVID-19- with CareMongering in Chatham-Kent | Chatham | ON | 3/17/2020 | 342 | 370 | 367 | 357 | 1.62 |
| Caremongers Stratford | Stratford | ON | 3/19/2020 | 328 | 581 | 722 | 761 | 5.16 |
| Creemore Caremongering | Collingwood | ON | 3/17/2020 | 324 | 409 | 449 | 481 | 2.44 |
| Caremongering Swift Current & Area: Community Response to COVID-19 | Swift Current | SK | 3/18/2020 | 322 | 357 | 363 | 369 | 2.80 |
| Caremongering- Midland/Penetang | Midland | ON | 3/19/2020 | 321 | 359 | 368 | 368 | 2.79 |
| Caremongering - Campbell River | Campbell River | BC | 3/26/2020 | 314 | 578 | 684 | 840 | 0.87 |
| Caremongering Greater Victoria | Victoria | BC | 3/17/2020 | 312 | 347 | 344 | 335 | 0.58 |
| CareMongering-OK: Kelowna & the Okanagan Community Response to COVID19 | Kelowna | BC | 3/15/2020 | 294 | 305 | 302 | 296 | 0.33 |
| Caremongering- Owen Sound: Owen Sound's Community Response to COVID-19 | Owen Sound | ON | 3/16/2020 | 287 | 339 | 343 | 352 | 1.18 |
| COVID-19 North Battleford and District Support | North Battleford | SK | 3/17/2020 | 279 | 307 | 305 | 300 | 2.28 |
| Thousand Islands Caremongering | Thousand Island Corridor (Serving Brockville- Kingston Corridor) | ON | 3/17/2020 | 275 | 293 | 302 | 298 | 1.02 |
| CareMongeringThePas | The Pas | MB | 3/17/2020 | 259 | 277 | 274 | 266 | 0.36 |
| Caremongering Midland Ontario- response time COVID 19 | Midland | ON | 3/20/2020 | 259 | 281 | 279 | 279 | 0.71 |
| CareMongering - GVRD - Greater Vancouver Community Response to COVID19 | Vancouver | BC | 3/16/2020 | 257 | 291 | 295 | 291 | 0.34 |
| CareMongering - Scarborough, ON: A Community Response to COVID19 | Toronto (Scarborough) | ON | 3/17/2020 | 257 | 336 | 394 | 475 | 1.49 |
| Caremongering Ottawa | Ottawa/Gatineau | ON | 3/17/20 | 241 | 272 | 274 | 271 | 0.74 |
| CareMongering - Fort St John Group | Fort St. John | BC | 3/16/2020 | 230 | 311 | 323 | 329 | 0.96 |
| Caremongering Woodstock: Community Response to COVID-19 | Woodstock | ON | 3/15/2020 | 223 | 234 | 232 | 114 | 1.28 |
| CareMongering - York Region: Markham/Cornell: Community Response to COVID-19 | Toronto (York Region) | ON | 3/16/2020 | 222 | 262 | 278 | 317 | 0.76 |
| caremongering Moncton - community response to covid-19 in Moncton NB | Moncton | NB | 3/13/2020 | 218 | 229 | 225 | 219 | 0.87 |
| CAREmongering-MONTREAL (Greater Montreal Area( | Montreal | QB | 3/18/2020 | 212 | 236 | 240 | 248 | 1.69 |
| Caremongering- Montreal: Response Communitaire a COVID-19 | Montreal | QB | 3/16/2020 | 211 | 272 | 314 | 319 | 3.68 |
| Care-Mongering NT: New Tech Community Response to Covid-19 | Alliston | ON | 3/28/20 | 207 | 257 | 260 | 254 | 0.39 |
| CareMongering Halton Hills (Acton and Georgetown) Community during COVID19 | Georgetown | ON | 3/17/2020 | 205 | 275 | 278 | 278 | 0.73 |
| Caremongering Neighbours (Peggy's Cove Rd & Prospect Rd. Communities) | Halifax (Peggys Cove) | NS | 3/16/2020 | 195 | 223 | 315 | 360 | 4.04 |
| Stouffville caremongering | Stouffville | ON | 3/17/2020 | 193 | 203 | 203 | 200 | 0.00 |
| Caremongering - Salmon Arm, BC | Salmon Arm | BC | 3/17/2020 | 193 | 200 | 198 | 196 | 0.50 |
| Caremongering- Calgary & Area | Calgary | AB | 3/16/2020 | 191 | 209 | NA | 210 | 0.48 |
| CareMongering - Penticton | Penticton | BC | 3/28/2020 | 174 | 207 | 209 | 218 | 0.97 |
| Windsor Caremongering or How We Stayed Together As A Community! | Windsor | ON | 3/18/2020 | 169 | 191 | 193 | 190 | 1.05 |
| CareMongering - City of Quinte West/Belleville/Trenton | Belleville | ON | 3/17/2020 | 169 | 187 | 191 | 188 | 1.60 |
| Caremongering Guelph- Community Support for our Difficult Reality | Guelph | ON | 3/23/2020 | 168 | 198 | 214 | 244 | 1.52 |
| Caremongering-YQR: Regina-Area Community Response to COVID-19 | Regina | SK | 3/16/2020 | 161 | 173 | 175 | 169 | 0.00 |
| Caremongering Medicine Hat | Medicine Hat | AB | 3/21/2020 | 152 | 161 | 160 | 156 | 0.62 |
| Caremongering - Meadowlands/Tiffany Hills #hamont | Hamilton | ON | 7/18/2018 | 145 | 148 | 148 | 147 | 0.68 |
| Care-mongering- Lanigan & Surrounding Area | Saskatoon (Lanigan) | Sk | 3/17/2020 | 141 | 151 | unable to locate | 149 | 0.66 |
| Caremongering - Eastern Ontario | Brockville (Eastern Ontario) | ON | 3/17/2020 | 139 | 144 | 143 | 141 | 0.69 |
| CareMongering - Vernon: Community Response To Covid-19 | Vernon | BC | 3/19/2020 | 137 | 143 | unable to locate | unable to locate | 0.70 |
| Caremongering York Region: COVID-19 | Toronto (York Region) | ON | 3/16/2020 | 133 | 183 | 201 | 208 | 3.83 |
| Alberni Caremongering | Port Alberni | BC | 3/21/2020 | 124 | 125 | 124 | 117 | 1.60 |
| CareMongering-Peterborough: PTBO Community Response to COVID19 | Peterborough* (Also Directed to Peterborough Shares) | ON | 3/14/2020 | 117 | 134 | 135 | 140 | 1.49 |
| Caremongering Woodstock ON/ Care Mongering Woodstock ON | Woodstock | ON | 3/18/2020 | 110 | 116 | 114 | 114 | 2.59 |
| Caremongering Portage la Prairie, Manitoba | Portage la Prairie | MB | 3/18/2020 | 109 | 117 | 118 | 129 | 0.85 |
| Caremongering- West Island & west region of Montreal | Montreal | QB | 3/17/2020 | 103 | 108 | 110 | 111 | 0.93 |
| Lean on Lethbridge-Caremongering- YQL Community Response to COVID-19 | Lethbridge | AB | 3/18/2020 | 93 | 94 | 93 | 92 | 0.00 |
| Caremongering Durham | Oshawa | ON | 3/15/2020 | 92 | 108 | 108 | 104 | 0.93 |
| Caremongering Prince Albert | Prince Albert | SK | 3/19/2020 | 87 | 90 | 86 | 84 | 0.00 |
| Caremongering St. Thomas- COVID-19 Volunteer Response Team | St. Thomas | ON | 4/1/2020 | 87 | 421 | 437 | 432 | 2.38 |
| Caremongering Winnipeg | Winnipeg | MB | 3/16/2020 | 79 | 89 | 89 | unable to locate | 1.12 |
| Caremongering - Nanaimo | Nanaimo | BC | 3/17/2020 | 78 | 85 | 86 | 87 | 1.18 |
| Community Support Low and Region/ Entraide communautaire low et region | Quebec City | QB | 4/3/2020 | 66 | 180 | 10 | 204 | 1.67 |
| CAREMONGERING BARRIE | Barrie | ON | 3/19/2020 | 52 | 55 | 54 | 54 | 3.64 |
| Caremongering - moncton-dieppe-riverview | Moncton | NB | 3/17/2020 | 50 | 53 | 53 | 53 | 0.00 |
| Caremondering Midland Ontario | Midland | ON | 4/3/2020 | 47 | 61 | 60 | 279 | 0.00 |
| Caremongering Montreal | Montreal | QB | 3/17/2020 | 47 | 48 | unable to locate | 489 | 0.00 |
| Caremongering Cathedral (Christ Church Cathedral, Fredericton) | Fredericton | NB | 3/22/2020 | 43 | 53 | 54 | 54 | 5.66 |
| Caremongering - Milton, ON | Milton | ON | 3/17/2020 | 37 | 37 | 37 | 37 | 0.00 |
| CareMongering Woodstock - Leaning into Community during COVID-19 | Woodstock | ON | 3/18/2020 | 22 | 23 | 23 | 22 | 4.35 |
| Caremongering Moose Jaw | Moose Jaw | SK | 3/17/2020 | 20 | 22 | unable to locate | 20 | 0.00 |
| Caremongering Vancouver | Vancouver | BC | 3/18/2020 | 17 | 33 | 36 | 43 | 3.03 |
| CareMongering Torbram - Brampton: Community Response to COVID-19 | Bolton (Torbram - Brampton) | ON | 3/26/2020 | 16 | 37 | 42 | 58 | 2.70 |
| Caremongering Bathurst New Brunswick - Community response to Covid-19 | Bathurst | NB | 3/23/2020 | 15 | 15 | 14 | 13 | 0.00 |
| Caremongering - London, Ontario, Canada | London | ON | 3/20/2020 | 9 | 9 | unable to locate | unable to locate | 11.11 |
| CAREmongering Nanaimo | Nanaimo | BC | 3/17/2020 | 4 | 4 | 4 | 4 | 25.00 |
| Caremongering Kelowna/West Kelowna | Kelowna | BC | 3/18/2020 | 1 | 1 | unable to locate | unable to locate | 0.00 |
| Caremongering Parksville | Parksville | BC | 3/17/2020 | 1 | 1 | 1 | 1 | 0.00 |
| Caremongering Brockville | Brockville | ON | 4/7/2020 | 0 | 22 | 22 | 22 | 0.00 |
| CareMongering- Warman | Warman | SK | 3/17/2020 | Not Available | Not Available | 279 | 276 |  |
|  |  |  |  |  |  |  |  |  |
| **TOTAL** |  |  |  | **175,276** | **194,879** | **179,356** | **192,665** |  |
